# A unique view on male infertility around the globe

**DOI:** 10.1186/s12958-015-0032-1

**Published:** 2015-04-26

**Authors:** Ashok Agarwal, Aditi Mulgund, Alaa Hamada, Michelle Renee Chyatte

**Affiliations:** Center for Reproductive Medicine, Cleveland Clinic, Cleveland, Ohio 44195 USA; Department of Urology, Jackson South Hospital, Miami University, Miami, FL 33176 USA; Northeast Ohio Medical University, 4209 State Route 44, PO Box 95, Rootstown, OH 44272 USA

**Keywords:** Male infertility, Global health, Fecundity, Worldwide

## Abstract

**Background:**

Infertility affects an estimated 15% of couples globally, amounting to 48.5 million couples. Males are found to be solely responsible for 20-30% of infertility cases and contribute to 50% of cases overall. However, this number does not accurately represent all regions of the world. Indeed, on a global level, there is a lack of accurate statistics on rates of male infertility. Our report examines major regions of the world and reports rates of male infertility based on data on female infertility.

**Methods:**

Our search consisted of systematic reviews, meta-analyses, and population-based studies by searching the terms “epidemiology, male infertility, and prevalence.” We identified 16 articles for detailed study. We typically used the assumption that 50% of all cases of infertility are due to female factors alone, 20-30% are due to male factors alone, and the remaining 20-30% are due to a combination of male and female factors. Therefore, in regions of the world where male factor or rates of male infertility were not reported, we used this assumption to calculate general rates of male factor infertility.

**Results:**

Our calculated data showed that the distribution of infertility due to male factor ranged from 20% to 70% and that the percentage of infertile men ranged from 2·5% to 12%. Infertility rates were highest in Africa and Central/Eastern Europe. Additionally, according to a variety of sources, rates of male infertility in North America, Australia, and Central and Eastern Europe varied from 4 5-6%, 9%, and 8-12%, respectively.

**Conclusion:**

This study demonstrates a novel and unique way to calculate the distribution of male infertility around the world. According to our results, at least 30 million men worldwide are infertile with the highest rates in Africa and Eastern Europe. Results indicate further research is needed regarding etiology and treatment, reduce stigma & cultural barriers, and establish a more precise calculation.

## Background

Infertility is a worldwide problem, and according to Sharlip et al, it affects 15% of couples that have unprotected intercourse [1]. Although this statistic is commonly cited, it is an amalgamation of numbers taken from around the world and thus does not reflect rates in specific countries and regions. On a global scale, accurate information regarding rates of male infertility is acutely lacking, and has not been accurately reported.

Calculating regionally based male infertility rates is challenging for a number of reasons. First, population surveys generally interview couples or female partners of a couple who have unprotected intercourse and wish to have children. This is a very specific population. As such, data from a significant number of infertile individuals is never included, which may bias the data.

Second, unlike female infertility, male infertility is not well reported in general but especially in countries where cultural differences and patriarchal societies may prevent accurate statistics from being collected and compiled. For example, in Northern Africa and Middle East, the female partner is often blamed for infertility. Men, therefore, do not usually agree to undergo fertility evaluation, resulting in underreporting of male infertility. Furthermore, polygamy is a common practice in many cultures [2]. One of the reasons for polygamy is to overcome infertility and increase the probability of having children. Additionally, in some African countries, the tradition of “Chiramu” allows an infertile male to bring in a brother or a relative to impregnate his wife [2]. In this way, the man retains his masculine identity and status in his community’s eyes.

A third challenge stems from the fact that male infertility has never been defined as a disease, which has resulted in sparse statistics. Additionally, demographic and clinical studies vary in epidemiological definition of infertility. While many clinical studies have examined infertility over the course of a year, several demographic studies examine infertility over a five-year projection [3]. Finally, while some studies only examine females, others only examine the men presenting to infertility clinics, which are generally small groups who are not representative of the larger population of infertile men.

Without accurate, region-specific data, it is not possible to identify and comprehensively treat infertile men. Therefore, to bridge this gap in knowledge, we have consolidated current data and, where recent information is lacking, estimated rates of male infertility using pre-existing data on female infertility in areas around the world. We focused especially on North America, Latin America and the Caribbean, North Africa and the Middle East, Sub-Saharan Africa, Europe, Eastern Europe, Central Asia, Eastern Asia, the Pacific, and Australia. The developing world has much less data available, which is why the above regions were selected.

Therefore, the goal of this commentary is to consolidate the large breadth of information available on male infertility and provide answers to the following two questions: How does the rate of male infertility vary in the different regions across the world? Can accurate estimates of male infertility be captured globally while identifying potential socio-economic and cultural reporting barriers that skew the results?

## Methods

**Figure 1:**
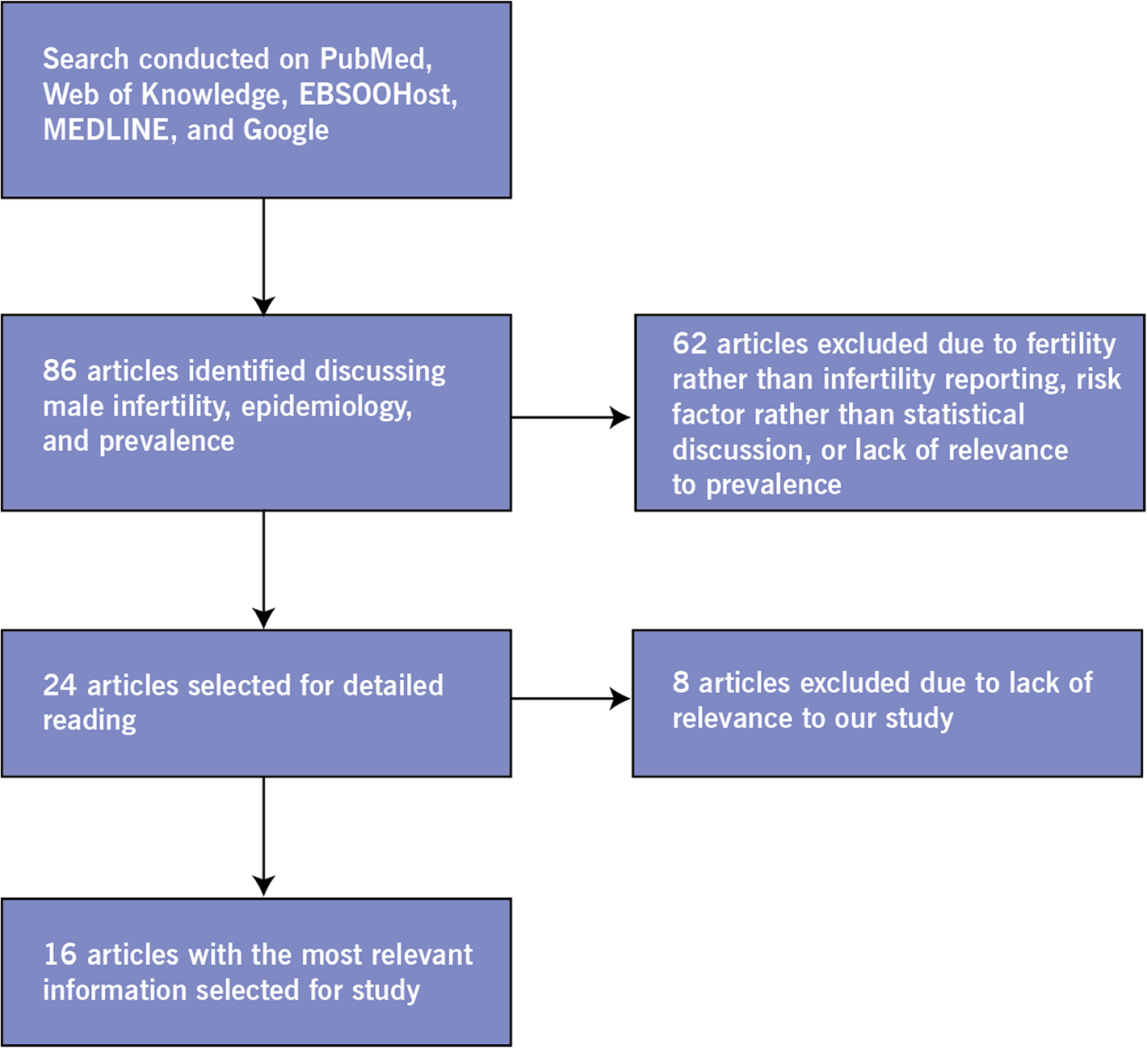
Flow chart demonstrating methodology. This figure is a representation of our methods, including number of articles found and filtered, and inclusion and exclusion criteria for the final article selection.

We limited our literature search to include only systematic reviews and meta-analyses (where possible) of mainly population-based studies. For factors that did not generate a result for meta-analysis or systematic review using the Boolean terms: “factor” AND systematic review” or “factor” AND meta-analysis,”, a search was done using that particular factor, e.g. “factor” AND male infertility” to elicit an original study that looked into the effect of that factor on any aspect of male infertility. We searched PubMed, Web of Knowledge, MEDLINE, EBSCOhost and Google Scholar using the following keywords: epidemiology, male infertility, and prevalence (Figure 1).

We initially identified 86 relevant articles in our multiple searches. Of those, 62 articles were excluded due to reporting of fertility rather than infertility, discussion of risk factors rather than statistics regarding overarching infertility, or lack of information regarding prevalence of infertility. From the remaining 24 articles, we further shortened the list to 16 articles that contained the most relevant information for our study. Most of these articles looked at a one-year definition of infertility. Mascarenhaas and colleagues used a 5-year demographic definition of male infertility in their study [3]. Our report uses the definition of infertility pertaining to one year. However, we report the numbers used by Mascarenhas and colleagues as well.

### Statistical analysis

It has been stated that 48.5 million couples that have unprotected intercourse suffer from infertility worldwide [4]. However, this statistic does not clearly define infertility by geographic region. Additionally, many clinical studies do not begin to explore infertility until a couple attempts to get pregnant for at least one year according to the World Health Organization (WHO). Demographic studies, on the other hand, look at infertility over a five-year projection [3]. In general, according to Sharlip, 50% of infertility cases are due to a solely female factor, pure male factor accounts for 20-30% of the problem, and the remaining 20-30% is due to a combination of both male and female factors [1]. We used the “Sharlip factor” as a basis for calculations because it was the most widely cited and reported statistic regarding male infertility. Further, a more accurate statistic is as of yet, unavailable. Therefore we used the same parameters to calculate the statistics found in this report. In regions where the prevalence of male infertility was not reported, we calculated male infertility statistics utilizing female infertility rates. This statistic was calculated by using the reported rate of infertility in that region. We applied Sharlip’s estimate that approximately 20-30% of the total infertile couple population could be attributed to male infertility [1]. We calculated percentages, such as each region’s total infertile male population. Using a combination of these two numbers, we were able to calculate an estimated number of infertile men. To further explain how our numbers were calculated, we provide an example. Data was taken from the WHO regarding infertility rates as reported by female partners in regions of the world. In Sub-Saharan Africa, 14.2% of women reported infertility. From this, we assumed with couples infertility at 14.2%, then female factor infertility would be 7.1%. Since the other 50% is assumed to be a combination of male factor and combined factor infertility, we calculated 20-30% of 7.1% to arrive at solely male factor infertility and 40-55% of 7.1% to arrive at any situation when the male factor is involved in any way.

## Results

The calculated global data shows that the percent of infertility that is attributable to males ranged between 20-70% (Table 1). Additionally, the percentage of infertile males in these countries varied from 2.5-12% (Table 1). The largest pockets of male infertility occurred in Central and Eastern Europe (8% to 12%) and Australia (8% to 9%). North America demonstrates rates of male infertility 4.5-6% [4]. While a calculated percentage reveals 4.5-6% of North American males are infertile, the Centers for Disease Control (CDC) estimates that 9.4% of males in the United States are infertile (Table 1) [4]. Sub-Saharan Africa is typically thought to have high rates of infertility; however, possibly due to underreporting, the rates shown in Table 1 appear low. The CDC and the WHO do not use the Sharlip calculation when reporting their data. Rather, their rates are based upon in-person interviews of representative populations, whereas Sharlip’s data is based upon previously reported data from a classic French study. What makes our data novel is the fact that we use the data that is representative of the population examined. For example, when calculating current infertility rates in North America, we use information from the CDC, which is representative of the North American population. However, this same data is not representative of the Sub-Saharan population. We therefore used female data from the WHO combined with the representative rates reported by Sharlip to calculate male infertility in those regions.

**Table 1:** **This table shows male infertility, based on various studies reporting male or female infertility globally**

	**Males that are reported infertile**	**Couples that are reported infertile**	**Couples in which the male factor is one of multiple factors involved**
**North America**	4.5-6%^a^	15%	50%
**Middle East**	Unknown	Unknown	60%-70%^b^ [23]
**Sub-Saharan Africa**	2.5%-4.8%^a^	12.5%-16% [22]	20-40% [22]
**Europe**	7.5%^a^ [11]	15% [11]	50% of all infertile couples
**Australia**	8%; 9%^b^ [10]	15%	40% [17]
**Central/Eastern Europe**	8%-12% [6,14]	20% [14]	56% [6]
**Asia**	Unknown	Unknown	37% [19]
**Latin America**	Unknown	Unknown	52% [19]
**Africa**	Unknown	Unknown	43% [19]

^a^Percentages were calculated from data reported on female infertility, using the assumption that 50% of infertility cases are due to females only, and 20-30% are due to male factor only.

^b^Study states that 60-70% of all men presenting to IVF clinics in the Middle East have some involvement in the cause of infertility.

Table 2 takes data from a WHO study conducted from 1994-2000. North and West Africa had the highest rates of infertility, which ranged from 4.24%-6.35%. Central and East Asia had the lowest rates of infertility, with 2·05%-3.07% of infertility cases due to male factor alone (Table 2). Cases of infertility due to both male and female factors ranged from 2.84% in Sub-Saharan Africa to 11.65% in Northern and Western Africa.

**Table 2:** **Calculated data taken from the WHO regarding infertile women, extrapolated to men, globally ranging from 1994-2000** [9]

	**Total Infertility Rate as reported by female partners (women who have had sexual intercourse but no pregnancy, ages 15-49)**	**Male factor only (20-30%); Female factor not involved**	**Male factor Involved (40-55%)**	**Female factor involved (50%)**
**Sub-Saharan Africa**	14.20%^a^ [18]	2.84%-4.26%^b,c^	2.84%-5.68% [19]	7.1%
**Central/East Asia**	10.23%^a^ [18]	2.05%-3.07%^b^	3.79% [19]	5.1%
**North/West Africa**	21.18%^a^ [18]	4.24%-6.35%^b^	8.47-11.65% [19]	10.6%
**Latin America/Caribbean**	13.70%^a^ [18]	2.74%-4.11%^b^	7.12% [19]	6.85%

^a^Female data reported by country; we used the mean of these countries’ data to define the region’s average reported infertility.

^b^Male data calculated based on the argument that while 50% of infertility is due to females, 20-30% is due to males. Specific male infertility rates for these regions are not well reported.

^c^Data calculated from a different source than Sub-Saharan Africa calculations in Table 1.

Table 3 shows infertility data in terms of regional populations. While most of our data was reported as percentages, we converted them into population absolute numbers in order to gain a broader understanding and perhaps more accurate estimate of the number of infertile men. The number of infertile men ranged from 5,000 to 18,000,000 with a worldwide estimate of 30,625,864 to 30,641,262 men who may be infertile. The highest number of infertile men was concentrated in Europe. According to this table, in any given region, at least 5,459 men may be infertile (Table 3).

**Table 3:** **This shows infertility reported as gross numbers, using global population estimates**

	**Total population of Region**	**Male population of Region**	**Total Male reproductive population (15-60y)**	**Male Infertility, %**	**Infertile Men**
**North America**	347,388,982	171,213,918 (49.2%)	116,254,250^a^	9.4% [4]	10,927,899
**Latin America/Caribbean**	582,418	287634 (49.3%)	361,099	Unknown	Unable to Calculate
**Sub-Saharan Africa**	850,000	420,000 (49.4%)	218,348	2.5%-4.8%	5,459-10,481
**Eastern Europe/Central Asia**	399,110	190,718 (47.8%)	259,421	8-12% [14]	20,754-31,130
**Europe**	734,228,972	353,542,772 (48.1%)	248,187,025^a^	7.5% [11]	18,614,027
**Asia/Pacific**	3,653,257	1,875,094 (51.3%)	1,199,437	Unknown	Unable to Calculate
**Oceania**	35,162,670	17,699,546 (50.3%)	11,752,499^a^	9% [10]	1,057,725

Explanation of calculations:

These numbers are crude estimations and calculations. They were calculated from two sources: UNFPA Country Profiles and World Stat. Both sources provided total population, male population, and population less than 15 years and greater than either 60 or 65 years. The calculations were performed as follows. For example, in Sub-Saharan Africa, the total population amounts to 850,000, according to UNFPA. The male population was 420,000. This amounts to 49.4% of the total population. The population less than 15 years old was 43% of the total population, and the population greater than 60 years old was 5% of the total population. These percentages convert to 365,500 and 42,500, respectively. After calculating that 49.4% of the total population is males, we also assumed that 49.4% of the total population between the ages of 15 and 60 were also males. Therefore, [850,000 – (365,500 + 42,500)] × 49.4% = 218,348. This is the total male reproductive population between the ages of 15 and 60. This number was multiplied by the percentage of male infertility prevalent in this population. 218,348 × (2.5% to 4.8%) = 5,459 to 10,481 infertile males present in Sub-Saharan Africa.

Unfortunately, some statistics were unable to calculate, due to the lack of reporting on these regions of the world.

^a^Population reported as Male, age 15-64.

Table 4 extrapolates male data from pre-existing female data reported in a systematic analysis conducted by the WHO. The rates of primary infertility, as reported by women, ranged from 1.5% to 2.6%, which were much lower than those reported over the course of 12 or more months. The male contribution to these rates of infertility ranged from 0.4% to 1.82% according to WHO estimates. Secondary infertility reported by women ranged from 7.2% to 18%, with the highest rates in Central and Eastern Europe, followed by South Asia at 12.2% and Sub-Saharan Africa at 11.65%. This data consolidated information between 1990 and 2010, providing a 5-year projection of infertility [3]. According to this data, the highest rates of infertility were concentrated through Africa and Central/Eastern Europe [3].

**Table 4:** **A 5 year extrapolation as reported by a Systematic Analysis of 277 Health Surveys on Female infertility** [11]

	**Primary infertility**		**Secondary infertility**	
	**Total 5 year infertility rate as reported by females** ^**a,b**^	**5 year Male factor infertility rate (calculated)**	**Total 5 year infertility rate as reported by females** ^**a,b**^	**5 year Male factor infertility rate (calculated)**
**Latin America**	1.5% [3]	0.78%^c^ [19]	7.5%	3.9%^c^ [19]
**North Africa/Middle East**	2.6% [3]	1.56-1.82%^c^ [23]	7.2%	4.32-5.04%^c^ [23]
**Sub-Saharan Africa**	2% [3]	0.4-0.8%^c^ [22]	11.6%	2.32-4.4%^c^ [22]
**Central/Eastern Europe**	2.2% [3]	1.23%^c^ [6]	18%	10.03%^c^ [6]
**South Asia**	2.2% [3]	0.81%^c^ [19]	12.2%	4.51%^c^ [19]
**East Asia/Pacific**	1.5% [3]	0.56%^c^ [19]	11%	4.07%^c^ [19]
**World**	1.9% [3]	0.38-0.57%^d^	10.5%	2.1-3.15%^d^

^a^Percentage of child-seeking women.

^b^Measured in 2010.

^c^Male data was calculated based on the various reported rates of male factor contribution to infertility cases in multiple studies (cited above).

^d^Male data for world was calculated based on the argument that while 50% of infertility is due to females, 20-30% is due to males.

Table 5 shows male infertility data (reported in earlier studies) from France, Western Siberia, Nigeria, Mongolia, Poland/Eastern Europe, Egypt, Iran, and Sudan [5-9]. We found that 6.4% to 42.4% of infertility cases in these areas were due to a male factor.

**Table 5:** **Infertility around the world** ^**a**^ **,** [12] **reported from previous studies examining male infertility to summarize previous research**

	**Population**	**Author, year**	**Female factor**	**Male factor**	**Combination**
**French Regions (1988-1989)**	1686 Couples	Thonneau et al. 1991 [13]	30%	20%	39%
**Western Siberia**	2000 Married women; 186 couples	Philippov et al. 1998 [27]	52.70%	6.40%	38.70%
**Southeastern Nigeria**	314 couples	Ikechebelu et al. 2003 [19]	25.80%	42.40%	20.70%
**Mongolia**	430 Couples	Bayasgalan et al. 2004 [28]	45.80%	25.60%	18.80%
**Poland/Eastern Europe**	Unreported	Sanocka and Kurpisz 2003 [14]; Bablok et al. 2011 [6]	Unreported	40-60%^b^ [14]; 56% [6]	Unreported
**Egypt**	190 Women	Inhorn, Buss 1994 [7]	82%	13%^c^; 46%^d^ [7]	Unreported
**Yazd Province of Iran**	5200 Couples	Aflatoonian et al. 2009 [8]	57.5%	25.3% [8]	8%
**Sudan**	710 couples	Elussein et al. 2008 [9]	49.3%	36.2% [9]	Unreported

^a^Table has been adapted from Winters and Walsh [12].

^b^This number was from Sanocka et al., which stated that 20% of couples are infertile, and 40-60% of those cases are due to male factor infertility [6]. This calculation amounts to 8-12% of men overall are the reason for these infertility cases.

^c^In Inhorn and Buss, in 11/87 (13%) of evaluated cases, male factor infertility was the sole cause of infertility [13].

^d^In Inhorn and Buss, in 40/87 (46%) of cases, male factor was involved [13].

## Discussion

### Global

**Figure 2:**
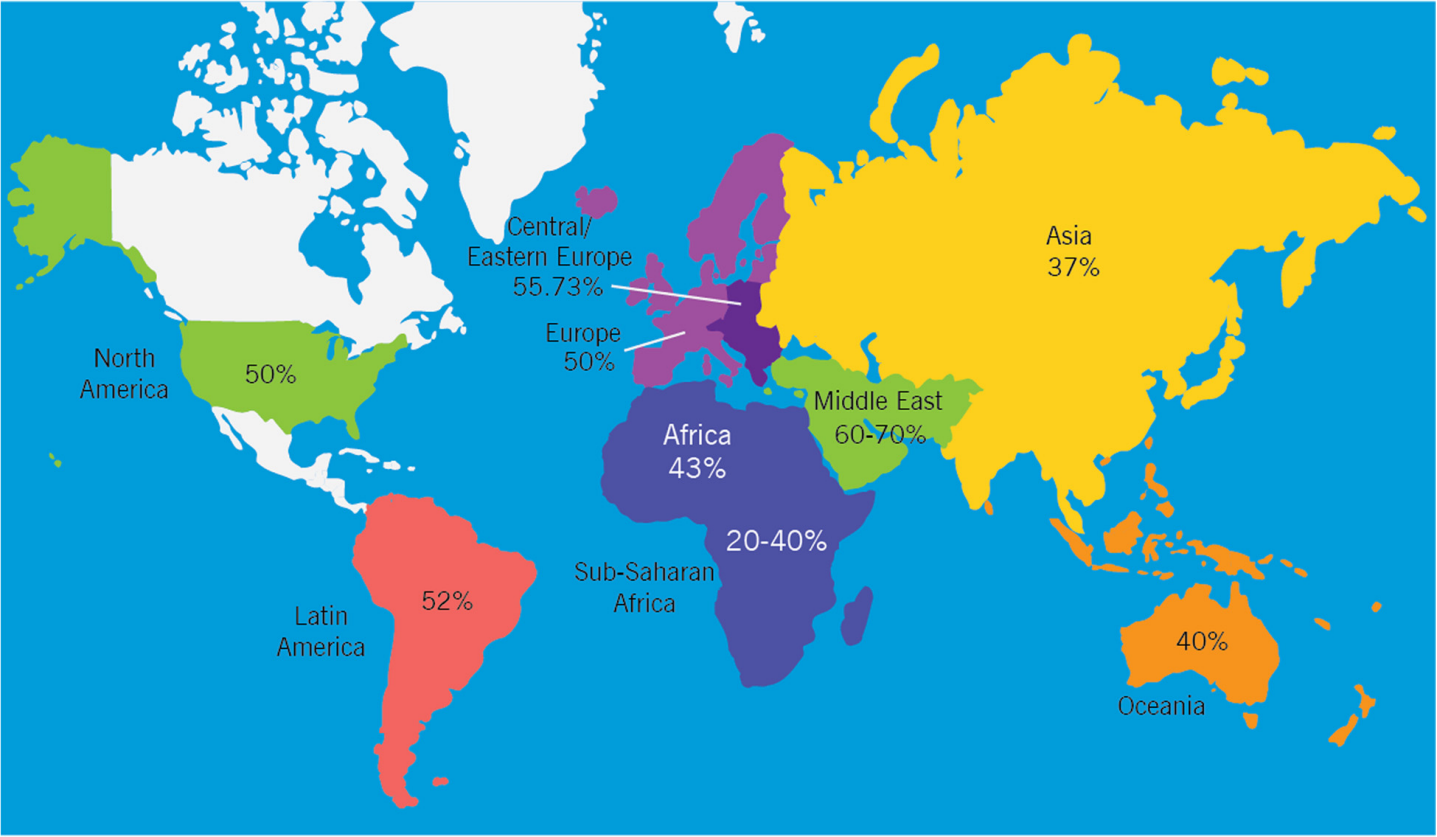
World map containing percentages of infertility cases per region that are due to male factor. This figure demonstrates rates of infertility cases in each region studied (North America, Latin America, Africa, Europe, Central/Eastern Europe, Middle East, Asia, and Oceania) due to male factor involvement.

Male infertility is a global population health concern. There are an estimated 48·5 million couples with infertility worldwide [3]. In the current study, we calculated rates of male infertility across the globe based on a review of the current literature (Figure 2). Since we do not know the actual rates of infertility, most of the numbers shown are based on self-report, thus cover a wide range. Overall, by examining the available literature and consolidating the information, our data indicates that global rates of male infertility range from 2.5% to 12%.

### North America and Europe

North America, Europe and Australia are developed countries, which may explain why rates of infertility are reportedly believed to be more accurate when compared to less developed countries. In North America, 4·5%-6% of males are infertile (Table 1). This number is similar to that of Australia, where 8% of males are infertile and 9% of males over the age of 40 have visited an infertility clinic at some point (Table 1) and Europe, where 7.5% of males are infertile. These numbers are based upon data from the National Health Statistics Report (NHSR) from the CDC National Health Interview Survey, the Australian Institute for Health and Welfare (AIHW), and the European Association of Urology (EAU) guidelines for male infertility [4,10,11]. These three regions of the world were the only organizations with the most accurate reporting of data available. The estimation that 20-30% of infertility is due to a sole male factor helped calculate numbers in the developing world, providing the most conclusive report of male infertility around the world. Quantifying the available information gives us insight into where the greatest need is for further research into underlying etiology and treatment.

When comparing regions with another, Europe reports similar population estimates as the United States, with 15% of European couples and 7.5% of men reported infertile [11]. Olsen and colleagues found that infertility varied across Europe. After 12 months, 51.1%, 43.2%, 37.9%, 19.1%, and 43.2% of couples sought help for infertility in Denmark, Germany, Italy, Poland, and Spain, respectively, with approximately 40% seeking help across the sample [12]. A classical French study completed by Thonneau and colleagues in 1991 examined 1686 infertile couples and found that in a small region of France to find that abnormal infertility was present in males 20% of the time, and present in females 34% of the time, and in both males and females 38% of the time [13]. This region is different from the whole of Europe, and statistics are sparse. However, Sanocka and colleagues state that Poland’s population is considered representative of Eastern Europe [14]. That study stated that 20% of couples are infertile in Poland, and 40-60% of those couples’ cases are due to male factor alone, whereas a more recent study by Bablok and colleagues states that 56% of infertility cases are due to an involved male factor [6,14]. The most interesting part of our manuscript references the fact that all these numbers reported are so different. We conclude that the large varieties in these numbers are largely due to cultural differences. In the United States and Europe, infertility is a problem that men often feel comfortable addressing with their physician. This allows the problem to both get addressed and reported statistically.

### Australia

We see that Australia’s rates are similar to those in North America and the United States, at 8-9%; additionally, 40% of infertility cases in Australia are due to male factor involvement (Tables 1 and 3; Figure 1) [10,15]. While the Australian Institute for Health and Welfares (AIHW) statistics data is on males aged 40 and older, the AIHW states that 8% of males have reported trying to have children unsuccessfully and 9% are being evaluated for infertility [10].

### Africa and the infertility belt

The rates in North Africa, Sub-Saharan Africa, and Eastern Europe are close to some of the higher percentages of male infertility estimated worldwide (Table 2) [16]. Male factor involvement for Table 2 was calculated using the statistics found by Cates, Farley, and Rowe in 1985 [17]. With the discovery that male infertility is most prevalent throughout this region, this may be where marketing for assisted reproductive therapy, treatment for infection, and efforts for WHO research can be concentrated.

The highest numbers relate to a region known as the “African Infertility Belt,” which stretches east to west across central Africa from Gabon to the United Republic of Tanzania [18]. This region of the world has very high rates of infertility in women, and as men are involved in up to 43% of the problem, the argument follows that male infertility is also high in this region [17,19,20]. Male factor contribution to infertility is also extremely high in the close geographical region of the Middle East [21]. We also noticed that primary infertility rates were much lower than secondary infertility. This may result from the high amount of child marriage and young pregnancy occurring in developing countries, and the later development of sexually transmitted diseases (STDs) and pelvic infections [22]. However, these numbers are of questionable significance due to the scant nature of their collection. Additionally, the population of sub-saharan Africa grows yearly. This does not imply that the rates of male infertility may not be high, but rather that the population may be growing in other ways. Typically, in regions of Africa and other societies, the male is seen as the dominant individual in both the community and the family structure. Therefore, men, especially in Africa and the Middle East do not report their infertility, as they believe it is emasculating to be unable to impregnate a woman. As a result of this, the men in these societies especially tend to blame females for the lack of child and do not get help.

### Other diseases

The “African Infertility Belt” also has high rates of STDs such as N. gonnorrhoeae and C. trachomatis, which may have some correlation and relationship with the high rates of infertility in this region of the world [23]. Collet and co-workers discovered that a tubal factor was present in 82.8% of females presenting to infertility clinics and frequently positive endocervical cultures for N. gonorrhoeae and C. trachomatis [23].

### Total absolute numbers calculated

We have drawn on the arguments that approximately 50% of cases are due to women, and 20-30% of cases are due to men. The remaining 20-30% of infertility cases is due to a combination of male and female factors. In Table 1, multiple reports state an infertility rate of anywhere from 2.5% to 12% [6,10,11,14,20,21]. Total numbers of infertile men worldwide may amount from 30,625,864 to 30,641,262 (Table 3). This number does not include estimates from Latin America or Asia (the most populous continent on the planet), due to underreporting there. These numbers indicate over 30 million more men and their female counterparts who could benefit from assisted reproductive technology (ART) and treatment for infertility. Additionally, regardless of the lower rates of infertility in North America, Europe, and Australia, these regions should not be neglected in the research for future treatment options. These regions also make up a part of the worldwide infertility phenomenon. While there may be regions of Africa and Asia attracting more urgent attention, this same consideration should be extended globally.

### Updated WHO guidelines

In 2010, the WHO changed their guidelines for semen analysis for the diagnosis of the infertile male [24]. In doing so, they established reference values that were much lower than their previous ones, resulting in more men qualifying as “normal” [6]. Now, a man with reference values of greater than 15 million sperm, greater than 5% normal morphology, and 40% progressive motility would be considered normal. [25] With the new guidelines, more men would be considered fertile, while there may be an unnoticed rise in the number of infertile men. Therefore, a recent study involving our group advises caution when interpreting the new WHO reference values because they have not yet been accurately defined to discriminate fertile from infertile men [25].

### Limitations of our study

One major limitation of our study is the number of infertile couples who have never participated in intercourse. Following this limitation, we therefore cannot estimate the number of infertile men who have never participated in unprotected sexual intercourse. Additional limitations of any epidemiological study regarding infertility and sexual activity include that the quality of data varies from very poor to very good. Reproductive information is private and couples may not be inclined to be truthful in surveys [26]. Many men may not be willing to participate in semen studies [26]. Another limitation included the difference between one-year infertility rates and the five-year infertility rates reported by Mascarenhas et al. [3]. This difference in rates over a five-year projection may be due to the fact that over five years, the cases of infertility may either resolve, these couples may have found an alternative to traditional conception, or the study could have suffered from attrition. A major limitation of this study is that much of our data are based on WHO studies from the 1900’s and that the definition of a male factor in these studies was not well defined. Male factor infertility was based on both abnormal semen analyses and on associated factors like varicoceles and urogenital infections, and STDs in men with normal semen analyses. In countries with an accurate registration of diseases, the prevalence of both male infertility and male factor leading to couples’ infertility is lower than that in developing countries. Rates from developing countries are more likely due to a problem with definition of male infertility and lack of accurate reporting rather than a true reflection of male infertility in those regions. Finally, the biggest limitation was that we based our calculations on Sharlip et al. and applied these numbers for female infertility to that of men.

## Conclusions

According to our results, at least 30 million men worldwide are infertile with the highest rates in Africa and Eastern Europe. However, due to the varying credibility and older nature of many of the articles analyzed, it is quite difficult to make a definite conclusion on the nature of these infertility rates.

The main message of these findings is that male infertility is a global health issue that has not been researched or studied to truly understand its magnitude and prevalence. This information provides insight into where the greatest need is for further research into underlying etiology and treatment. The major recommendations of this manuscript are:

1. As a society, we must reduce barriers from stigmas associated with infertility due to religious and cultural beliefs.
2. We must create a globally accepted population-based calculation in order to understand the prevalence and magnitude of male infertility.
3. Much work is needed to raise awareness about male infertility.

With broad and accurate understanding, we can both treat infertility by managing underlying conditions.

## Abbreviations

ART: Assisted reproductive technology
AIHW: Australian institute for health and welfare
CDC: Centers for disease control
EAU: European association of urology
NHSR: National health statistics report
STDs: Sexually transmitted diseases
WHO: World Health Organization
